# Dihydrotestosterone, and Not Testosterone, Enhances the LPS-Induced Inflammatory Cytokine Gene Expression in Human Adipocytes

**DOI:** 10.3390/biomedicines11041194

**Published:** 2023-04-17

**Authors:** Angelo Di Vincenzo, Marnie Granzotto, Marika Crescenzi, Vincenzo Vindigni, Roberto Vettor, Marco Rossato

**Affiliations:** 1 Internal Medicine, Department of Medicine, University-Hospital of Padova, 35128 Padova, Italy; marnie.granzotto@unipd.it (M.G.); marikacrescenzi@hotmail.com (M.C.); roberto.vettor@unipd.it (R.V.); marco.rossato@unipd.it (M.R.); 2 Plastic and Reconstructive Surgery Unit, Department of Neurosciences, University-Hospital of Padova, 35128 Padova, Italy; vincenzo.vindigni@unipd.it

**Keywords:** adipose tissue, testosterone, adipocytokines, inflammation

## Abstract

Background: The development of obesity-related complications lies in the low-grade inflammatory state consequent to adipocyte dysfunction. The direct involvement of sex hormones in adipose tissue inflammation has been previously suggested, but the evidence is scarce. In this study, we evaluated the effects of sex steroids on the in-vitroexpression of inflammatory mediators in human-derived adipocytes before and after lipopolysaccharide (LPS) exposure. Methods: Human adipocytes were differentiated from the vascular stromal fraction of adipose tissue samples of subjects undergoing abdominoplasty. We evaluated MCP-1, IL-1β, IL-6, and TNF-α gene expression in the presence of the main sex steroids, testosterone (T), and 17β-estradiol (E). Furthermore, we analyzed the effects of adipocytes exposure to the non-aromatizable androgen dihydrotestosterone (DHT), together with the effects of adipocytes pre-incubation with the aromatase inhibitor anastrozole alone (A), and in combination with T (A/T) before incubation with LPS. Results: DHT, but not T, significantly enhanced the LPSinduction of MCP-1, IL-1β, IL-6, and TNF-α. Intriguingly, the exposure of adipocytes with A/T dramatically increased the LPS-induced expression of all considered inflammatory cytokines, even more than a hundred-fold. Conclusions: DHT and A/T dramatically enhance LPS-induced inflammatory cytokine expression in human-derived adipocytes. These results confirm the involvement of sex hormones in adipose tissue inflammation, suggesting a specific role for non-aromatizable androgens as the amplificatory sex hormones of the inflammatory response.

## 1. Introduction

Obesity is a condition with a high clinical impact due to associated complications such as type 2 diabetes mellitus (T2DM) and cardiovascular diseases (CVDs). It is defined by the expansion of adipose tissue, during conditions of energy excess, via processes of adipocyte hyperplasia and hypertrophy. However, along with fat mass enlargement, adipocytes become dysfunctional, developing biochemical and metabolic abnormalities [1]. In addition, after appropriate stimuli, adipocytes may act as somehow “immune” cells, activating complement components, recruiting macrophage [2], producing inflammatory cytokines that reach the circulatory system, thus promoting a systemic chronic low-grade inflammatory state [3,4]. High concentrations of these inflammatory cytokines inadipose tissue have been positively related toinsulin resistance, andthe development of obesity-related complications probably lies in this biological pro-inflammatory response as confirmed also by the promising results obtained with anti-inflammatory drug therapy [5,6].

Concomitantly, obesity is frequently characterized by altered sex hormone plasma levels and reproductive function abnormalities. About 45% of men suffering from moderate–severe obesity present hypogonadotropic hypogonadism [7,8], and severe obesity is associated with ovarian dysfunction in 36% of women [9]. Furthermore, in males, it has been observed an inverse relationship between BMI, waist circumference, and testosterone plasma levels. These conditions should be pointed out in clinical practice, considering the involvement of sex hormones in the modulation of metabolic and cardiovascular risk. In fact, in males, reduced testosterone levels have been associated with the development of T2DM and CVDs [10], and testosterone supplementation in hypogonadal patients seems to reduce this risk [11]. The exact role that sex hormone abnormalities play in CVDs development has been speculated for a long time.It is well known that the incidence of CVDs in adults is higher in men than women and that the CVD risk further increases with age in men and after menopause in females, causing the loss of estrogensprotective effect. Furthermore, circulating testosterone and inflammatory cytokine have been reported to be inversely related in the general population. On the contrary, testosterone replacement therapy (TRT) has been associated with the improvement of inflammatory parameters in hypogonadal men, along with body weight reduction [12,13,14]. In this regard, weight loss treatments in obesity usually promote an increase in androgens and a concomitant reduction in inflammatory cytokine plasma levels [15]. On the other hand, androgen-deprivation therapy, usually proposed to patients with metastatic prostate cancer, is associated with weight gain and the risk of developing T2DM. Finally, adipose-derived cytokines directly affect reproductive function, negatively influencing gonadal activity and the hypothalamic regulation of gonadotropin-releasing hormone secretion.

These data support the existence of a crosstalk between fat mass, inflammatory cells and sex hormones, which probably depend also in the expression of androgen and estrogen receptors in adipocyte [16,17], together with the peculiar enzymes of the adipose tissue such as aromatase and 5α-reductase, catalyzing the transformation of testosterone (T) to 17β-estradiol (E) and to dihydrotestosterone (DHT), respectively [18,19]. The expansion of fat depots and the consequent central obesity might magnify this enzymaticprofile. Thus, it has been proposed that sex steroids might directly and systematically modulate the inflammatory response within the adipose tissue depots, although the evidence about this issue is still limited.

In the present study, we evaluated the effects of T, E, and DHT on the expression of the adipocytokines MCP-1, IL-1β, IL-6, and TNF-α in human-derived adipocytes before and after stimulation with lipopolysaccharide (LPS), defining the possible involvement of sex hormones in the “immune response” of the adipose tissue.

## 2. Materials and Methods

### 2.1. Adipocytes Isolation and Differentiation

Subcutaneous adipose tissue (SAT) was obtained from 5 healthy male subjects, not taking any drugs, undergoing plastic surgery for abdominal wall laxity after weight loss by bariatric surgery (mean BMI 36.2 ± 7.3). Informed consent was obtained from all studied subjects.

The stromal vascular fraction was isolated from SAT by collagenase type II digestion (1mg/mL; Sigma–Aldrich, St. Louis, MO, USA) at 37 °C for 1 h and seeded in DMEM/F12 supplemented with 10% fetal bovine serum (0.35 × 10^6^ cells per well in 24-well plates). The cells were placed in a humidified incubator at a temperature of 37 °C and in a 5% *v*/*v* CO_2_ atmosphere. After 16–20hof cell adhesion, a serum-free adipogenic medium containing DMEM/F12 supplemented with 33 μmol/L of biotin, 17 μmol/L of pantothenate, 10 μg/mL transferrin, 66 nmol/L of insulin, 100 nmol/L of dexamethasone, 1 nmol/L of triiodothyronine, 0.25 mmol/L of 3-isobutyl-1-methylxanthine (IBMX), and 10 μmol/L of rosiglitazone was added to the cultures. After three days, the medium was replaced with adipogenicmedium without IBMX and rosiglitazone. The adipogenic medium has been replaced three times a week until the complete differentiation into mature adipocytes.

**Figure 1:**
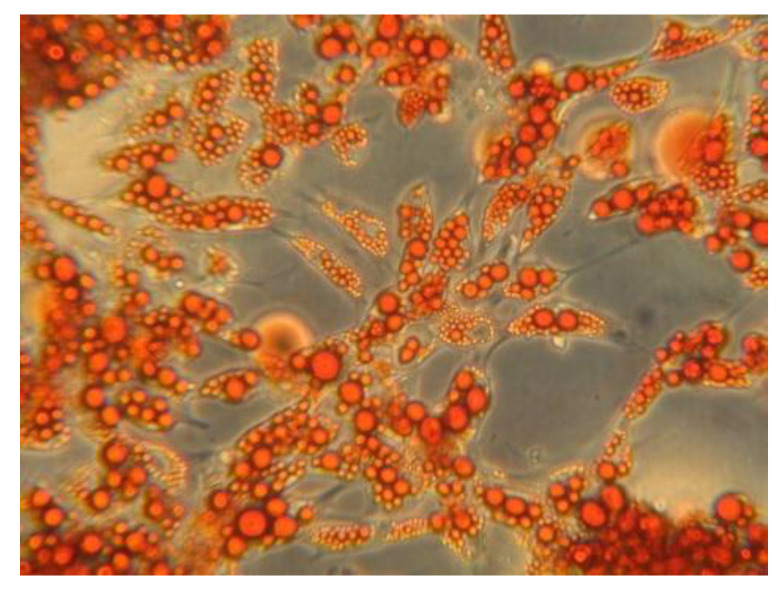
Oil-red-O staining of mature human adipocytes differentiated in-vitro from stromal vascular fraction displaying cytoplasmic lipid droplets.

The differentiation of the vascular-stromal component in the adipogenic medium was verified by oil-red-O staining, which is used to identify neutral lipids, in particular triglycerides (Figure 1), and by the gene expression of peroxisome proliferator-activated receptor γ (PPARγ), leptin, and fatty acid binding protein-4 (FABP-4), which are typically expressed in mature adipocytes [20].

### 2.2. Grouping

Fully differentiated adipocytes were stimulated overnight with 100 nmol/L testosterone (T group), 17 β-estradiol (E group), 5α-dihydrotestosterone (DHT group), and 200 nmol/L anastrozole (A group), alone or in combination 2 ng/mLLPS 4 h after each sex hormone treatment. A control group was incubated with the vehicle dimethyl sulfoxide (DMSO). Testosterone, 17 β-estradiol, 5α-dihydrotestosterone, anastrozole, and LPS were of the highest grade of purity that was commercially available from Sigma-Aldrich. A complete set of six experiments for each treatment was performed from each subject and run in triplicate. 

### 2.3. RNA Extraction and Real-Time PCR Gene-Expression Analysis

After 24 h of treatment, each group of mature adipocytes was washed with PBS buffer and RNA was extracted using a specific kit (RNEasy Kit, Qiagen GmbH, Hilden, Germany). The amount of RNA recovered by eluting the column with Rnase-free water was evaluated with a spectrophotometer, while the quality of the same was evaluated by a bioanalyzer (Agilent 2100, Santa Clara, CA, USA). First-strand cDNAs were synthesized from equal amounts of total RNA using random primers and M-MLV reverse transcriptase (Promega Italia, Milan, Italy). The cDNA was used to quantify gene-expression levels of adipocytokines using SYBR green fluorophore. The change in fluorescence at every cycle was monitored, and a threshold cycle above the background for each reaction was calculated. A melt curve analysis was performed following every run to ensure a single amplified product for every reaction, and all reactions were carried out and at least duplicated for every sample. Relative mRNA transcript levels were quantified with the 2^–∆∆ct^ method using Ribosomal Protein Lateral Stalk Subunit P0 (RPLP0) as a housekeeping internal control gene.

### 2.4. Statistical Analysis

Statistical analysis was performed using GraphPad Prism software. Results from five separate experiments were considered and expressed as mean values ± standard deviation (SD). Comparisons of data from two groups were analyzed with Student’s t-test. Comparisons of data from multiple groups were conducted byANOVA analysis. Differences between groups were considered statistically significant at *p* ≤ 0.05.

## 3. Results

### 3.1. DHT Exposure Reduces the Spontaneous Gene Expression of IL-6 and TNF-α in Adipocytes

**Figure 2:**
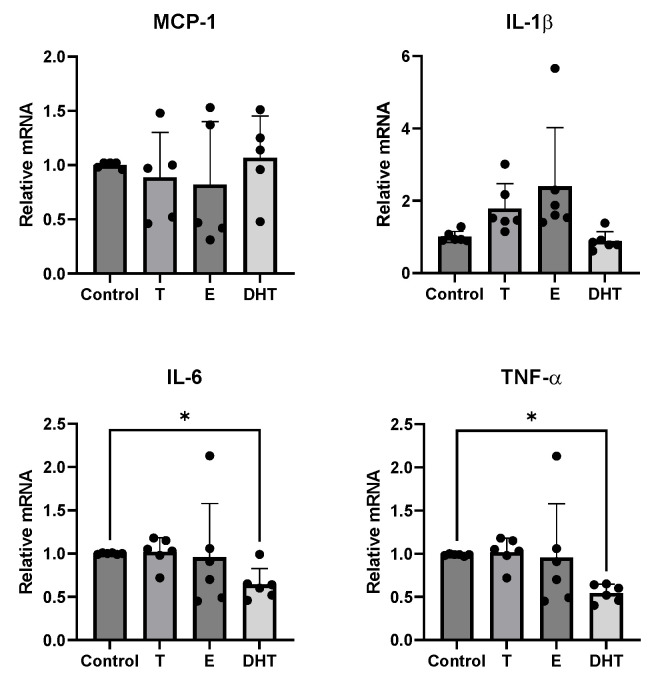
Restinggene expression of inflammatory cytokines (MCP-1, IL-1β, IL-6, and TNF-α) in human adipocytes in the presence of testosterone (T), 17 β-estradiol (E), and dihydrotestosterone (DHT). Control adipocyte cultures were incubated in the presence of vehicle only. A complete set of six experiments for each treatment was performed from each subject and run in triplicate. Data are expressed as mean ± SD. Each black dot represents the mean of triplicate measurements of a single experiment. * *p* < 0.05 with respect to control group.

Human differentiated adipocytes were incubated in the presence of T, E, DHT, or with vehicle only as control. As reported in Figure 2, neither T nor E affected the MCP-1, IL-1β, TNF-α, and IL-6 gene expression from human adipocytes at baseline. On the contrary, DHT exposure negatively modulated the basal expression of inflammatory cytokines, reaching statistical significance for IL-6 and TNF-α (*p* < 0.01).

### 3.2. DHT Increases the LPS-Induced Gene Expression of MCP-1, IL-6, and TNF-α in Human Adipocytes

**Figure 3:**
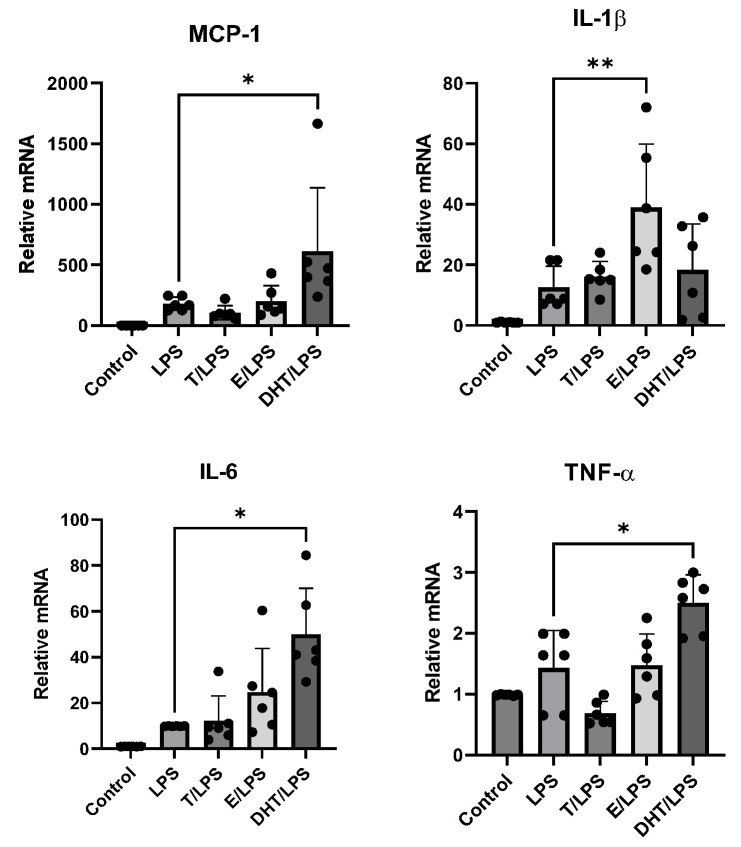
LPS-stimulated expression of inflammatory cytokine genes(MCP-1, IL-1β, IL-6, TNF-α in human adipocytes in the absence and presence of testosterone (T), 17 β-estradiol (E), and dihydrotestosterone (DHT). Control cultures were incubated in the presence of vehicle only. A complete set of six experiments for each treatment was performed from each subject and run in triplicate. Data are expressed as mean ± DS. Each black dot represents the mean of triplicate measurements of a single experiment. * *p* < 0.001; ** *p* < 0.05.

Human adipocytes were stimulated with LPS alone or after pre-incubation with T, E, and DHT. LPS incubation promoted an increase in adipocyte gene expression of all considered inflammatory cytokines with respect to the control condition. T exposure did not affect adipocytokine expression after the inflammatory stimulus. On the contrary, E showed a trend to enhance inflammatory cytokine expression when combined with LPS, although the statistical significance was reached only for IL-1β (*p* < 0.05). Interestingly, after pre-incubation with DHT, LPS induced an important and significant amplification of mRNA expression of MCP-1, IL-6, and TNF-α (*p* < 0.001, Figure 3). These significant amplificatory effects were not observed for IL-1β (Figure 3).

### 3.3. Anastrozole Exposure Amplifies the LPS-Induced Adipocytokines Expression in Adipocytes

Since only DHT but not T promoted inflammatory cytokine expression in human adipocytes after LPS exposure, we explored if this effect was peculiar only for non-aromatizable androgens. Thus, we tested the effects of the aromatase inhibitor anastrozole (A) on the inflammatory cytokine gene expression in human adipocytes in-vitro.

**Figure 4:**
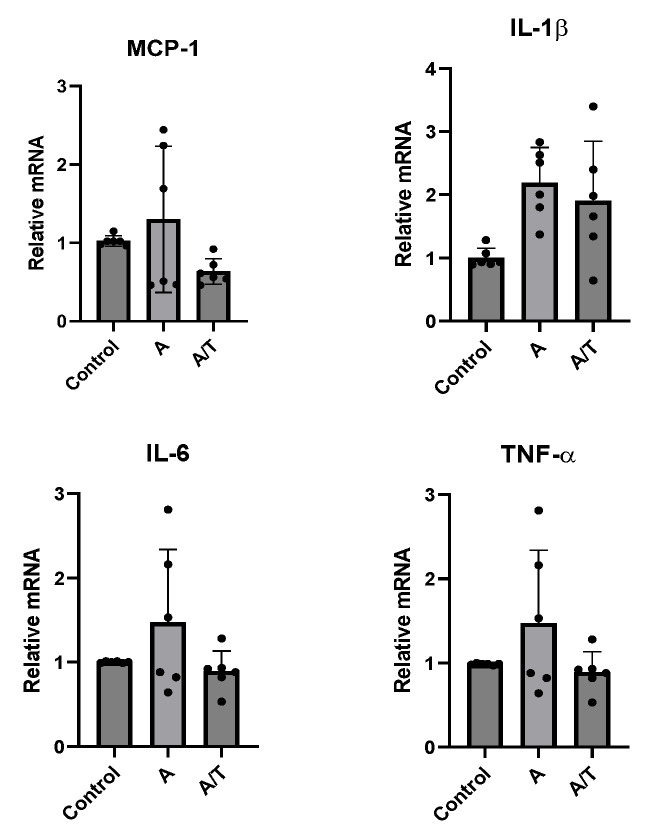
Gene expression of inflammatory cytokines (MCP-1, IL-1β, IL-6, and TNF-α) in human adipocytes in the presence of anastrozole alone (A) and in combination with testosterone (A/T). Control cultures were incubated in the presence of vehicle only. A complete set of six experiments for each treatment was performed from each subject and run in triplicate. Data are expressed as mean ± DS. Each black dot represents the mean of triplicate measurements of a single experiment.

At baseline, no significant effect was observed after treatment with A alone or together with T (Figure 4). 

**Figure 5:**
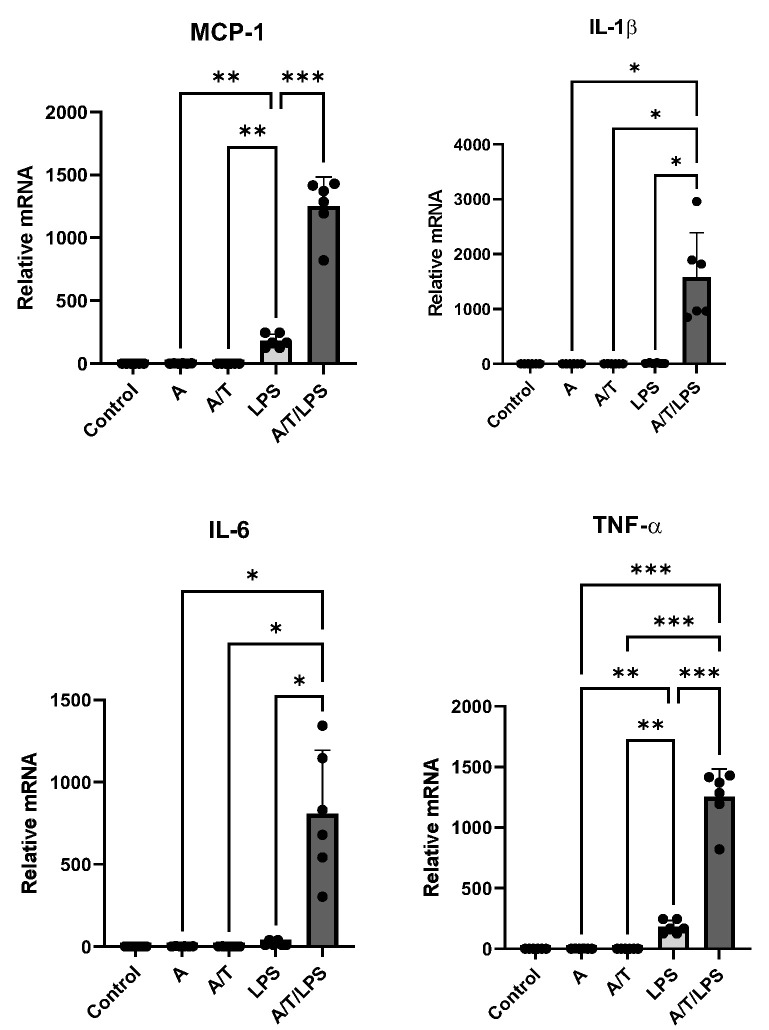
Gene expression of inflammatory cytokines (MCP-1, IL-1β, IL-6, and TNF-α) in human adipocytes in the presence of LPS alone and in combination with T (A/T/LPS). Control cultures were incubated in the presence of vehicle only. A complete set of six experiments for each treatment was performed from each subject and run in triplicate. Data are expressed as mean ± DS. Each black dot represents the mean of triplicate measurements of a single experiment.* *p* < 0.05; ** *p* < 0.001; *** *p* < 0.005.

However, a marked, even more than a hundred-fold, increase in the relative expression of all considered cytokines (MCP-1, IL-1β, IL-6, and TNF-α) was observed when adipocytes were exposed to LPS after pre-treatment with A/T (*p* < 0.001, Figure 5).

## 4. Discussion

Both pre-clinical and clinical studies reported the role of androgens in adipogenesis and confirmed sexual dimorphism in response to testosterone stimulation [21].

The development of android obesity, characterized by abdominal fat deposition, is a common feature of obesity in males and represents one of the main cardiovascular risk factors. In the vicious cycle in which central obesity promotes reduced testosterone levels while hypogonadism favors fat tissue deposition, the consequent imbalance in inflammatory cytokine production may represent a missing link with respect to the development of T2DM and CVDs in affected patients. In fact, adipose tissue acts as an endocrine organ, producing mediators involved in metabolic homeostasis and vascular health. Furthermore, evidence from clinical research suggeststhe possible anti-inflammatory action of testosterone during the treatment of male hypogonadism.

In-vitro evidence showing the effects of sex hormones on the expression of inflammatory mediators in adipocytes is scarce and sometimes conflicting. A study by Su et al. in 2014 reported that, in 3T3-L1 adipocytes, testosterone enhanced the LPS-induced expression of IL-6 and MCP-1 [22]. However, a previous study proposed an anti-inflammatory effect of DHT on 3T3-L1 adipocytes [23]. On the other hand, in animal models, estrogens showed more anti-inflammatory potential than androgens, and they seemed to prevent the detrimental effects of a high-fat diet by providing a reduction in the plasma and white adipose tissue concentrations of TNF-α and IL-6 [24]. The dysfunctional adipose tissue secretome has primary relevance in the development of obesity-related complications. In particular, adipose-derived inflammatory mediators seem to promote the development of insulin resistance and affect endothelial function, leading to both T2DM and CVDs in obesity. High sensitivity C-reactive protein is currently used as a risk biomarker in overt atherosclerosis, but also TNF-α seems to be involved in the systemic inflammatory response, leading to vascular dysfunction and atherosclerotic plaque instability. MCP-1 plays a direct role in the inflammation of adipose tissue and the activation of resident macrophages, and higher circulating levels are commonly observed in patients with obesity with respect to the lean counterpart [25]. Similarly, IL-1β release is enhanced in the visceral depot in obesity [26]. The role played by IL-6 seems to be even more complex; besides its inflammatory action, it also seems to be involved in the modulation of exercise effects on adipose tissue [27].

Despite the main limitations (in-vitro data only from SAT biopsies), the present study shed new light on the relationships between sex hormones and the “inflammatory response” of adipose tissue. In particular, we confirmed the pro-inflammatory role of androgens but we observed that, in human derived-adipocytes, this is true only for non-aromatizable androgen. In fact, in human adipocytes, the incubation with DHT, a non-aromatizable testosterone form, but not with T increased the expression of MCP-1, IL-6, and TNF-α after stimulation with LPS. In addition, we also reported for the first time that the inhibition of aromatase with anastrozole exaggeratedly increased the pro-inflammatory effect of T on the LPS-induced expression of MCP-1, IL-1β, IL-6, and TNF-α in human adipocytes. The chronic, low-grade, inflammatory state characterizing obesity is sustained by adipokines and other proinflammatory mediators released locally in the adipose tissue. Thus, the identification of the mechanisms possibly involved in the modulation of their synthesis and release is of primary importance.

Testosterone supplementation in subjects with low androgens plasma levels has been associated with a reduction in inflammatory markers; despite this, its use remains controversial due to the risk of developing different systemic complications such as erythrocytosis, CVDs, and lower urinary tract symptoms together with prostate hyperplasia and cancer [28,29].

The results of our study suggest that this opposite effect may be attributed to the peculiar enzymatic profile of adipose tissue: in fact, the presence of aromatase and 5α-reductase in adipocytes, promoting the peripheral conversion of circulating T to E and T to DHT, respectively, may influence the individual response to androgens, affecting the relative concentration of the biologically active form. Furthermore, a possible modulatory effect of the different expressions of adipose tissue androgen and estrogen receptors has to be considered, due to their role in adipogenesis and adipocyte commitment [30].

However, at this moment, the evidence to support our hypothesis is very limited. Aromatase is expressed in different cell types, such as adipocytes but also carcinoma and intra-tumoral stromal cells, in particular in breast cancer. Aromatase inhibitors are used in clinical practice only in women, in the context of hormone-receptor-positive breast cancer, but it is interesting to note that about half of treated patients develop a peculiar form of arthralgia, whose underlying mechanism remains unclear. In mice, the administration of letrozole, another aromatase inhibitor, has been associated with increased circulating levels of IL-6 [31].

In addition, in animal models of insulin resistance, increased aromatase activity has been demonstrated to be associated with the reduced expression of MCP-1 and TNF-α [32], thus supporting a role for this enzyme in the regulation of the systemic inflammatory response.

Even lesser evidence is available on the effect of 5α-reductase modulation of the inflammatory response. Previous studies reported an increased risk of developing non-alcoholic fatty liver disease and T2DM during treatment with 5α-reductase inhibitors [33], probably due to the relative hypogonadal state with reduced DHT levels.

Finally, our results could be in line with the clinical observation of a less severe outcome in male patients with obesity during infections. In fact, this so called “obesity paradox” has been observed in sepsis, with higher BMI associated with better survival rates with respect to matched normal-weight subjects [34]. To justify this clinical paradox, several studies proposed a protective effect of obesity in sepsis [35,36,37], but a plausible underlying mechanism was not recognized. Sepsis is characterized by a dysregulation of pro-inflammatory and anti-inflammatory processes with a consequent exaggerated host response to pathogens, and epidemiological data show that sepsis is more frequent in males [38]. From an evolutionary point of view, inflammation is a protective, time-dependent mechanism of the organism in response to a danger signal, but it may become counterproductive in the case of long-lasting stimulations or, as in sepsis, because of an uncontrolled acute inflammatory response. The development of an inflammatory state, though of a different extent, might represent a common feature linking obesity and sepsis, but with a suppressive rather than additive effect. According to our data, the low testosterone plasma levels in male patients with obesity might have an additional protective role, limiting the expression and release of inflammatory cytokines.

Another relevant limitation of our study lies in the availability of only gene expression, without data on cytokines secretion. However, although preliminary, thesefindingsseem to supporta tight connection between sex hormone imbalance and inflammatory response.

## 5. Conclusions

Adipose tissue is involved in the systemic inflammation that characterizes obesity, and sex steroids seem to modulate this activity. Here, we demonstrated that the in-vitro exposure of human adipocytes to testosterone only in its non-aromatizable form is associated with an increased expression of different inflammatory cytokines in these cells. 

In this regard, the increased aromatase expression and activity characterizing adipose tissue in obesity could have protective effects in some clinical pro-inflammatory conditions. In particular, this could be in line with clinical observations suggesting an “obesity paradox” in sepsis, with lower mortality in patients with obesity compared to lean subjects probably related to a less pronounced inflammatory response, above all in male subjects. Morestudies are needed to confirm these hypotheses, and even to evaluate thepossible negative effects of androgen-replacement therapy in obese hypogonadal patients during infectious diseases.

## Data Availability

The datasets used and analyzed during the current study are available from the corresponding author upon reasonable request.
